# Customer pressure and environmental stewardship: The moderator role of perceived benefit by managers

**DOI:** 10.1371/journal.pone.0306616

**Published:** 2024-07-05

**Authors:** Vo Tan Liem, Nguyen Ngoc Hien

**Affiliations:** 1 Faculty of Accounting and Finance, Van Hien University, Ho Chi Minh City, Vietnam; 2 Faculty of Business Administration, Industrial University of Ho Chi Minh City, Ho Chi Minh City, Vietnam; Swinburne University of Technology - Sarawak Campus, MALAYSIA

## Abstract

Nowadays, environmental issues and cleaner products are interest to many customers, considering whether to buy or continue using a product. This will affect the perception, attitude of upper managers in the process of strategic choices and operational management behavior. This study is based on the Upper Echelon Theory, research under the influence of customer pressure to attitude toward the environmental, decision choices for cleaner production strategies, implementation of environmental management accounting towards achieving green competitive advantage of Vietnamese manufacturing enterprises. In addition, the role of two moderator variables: (1) perceived benefit of cleaner production strategies and (2) perceived benefit of environmental management accounting in the research model is also considered. This study surveyed 234 CEOs of Vietnamese manufacturing enterprises. This study employed PLS-SEM, version 3 for data analysis. Results have shown that all relationships are statistically significant. Moderator variables have a statistically significant and positive impact in relationships in which they play a moderator role. This study helps CEOs realize the importance of producing products that are customer-oriented, environmentally friendly, and the implementation of environmental management accounting will have a strong impact on achieving a sustainable competitive advantage.

## 1. Introduction

Questionable environmental practices by firms lead to decreased customer demand for their products [1]. Both internal and external factors have an impact on how firms respond to environmental challenges [2]. Yang et al. [3] stress the importance of taking into account all stakeholders in environmental decision-making. External pressures are starting to influence how top executives within the firm are seen [2]. Consumers consistently push companies to develop in ways that minimize their effect on the natural environment [3]. Studies have been undertaken in multiple nations to examine how managers’ environmental views impact their decision-making processes. However, research on attitude toward the environment is scarce. Studies have analyzed environmental attitudes in many countries such as Japan, Sweden, Turkey, Canada, the Caribbean, and China, linking them to variances in environmental performance [4]. The process of economic reform, which started in 1986, aimed to transform the centrally planned economy into a market economy with a socialist orientation, and it is still ongoing [5]. A multitude of novel activities and enterprises have surfaced, with environmental challenges garnering significant attention. Thus, within the framework of a country facing resource depletion, escalating environmental pollution, and deteriorating climate change, etc…, the majority of reports and researches have recognized the significance of a green economy, green production and cleaner production, which is an unavoidable trend in the future [6].

Empirical studies on the psychological aspects of managers’ attitude toward the environmental in developing countries, particularly Vietnam with a transitional economy, have been scarce [7]. CEOs’ psychological traits, such as their attitude toward the environmental, have not been given much focus, despite their potential to help firms become more environmentally friendly and gain green advantages. Furthermore, there is a need for deeper exploration of psychological traits that are translated into strategic decision-making, as well as the design and implementation of control systems concerning environmental issues and performance [8, 9].

Top managers’ environmental attitudes are expected to impact the selection of cleaner production strategies, the implementation of environmental management accounting, and the development of green competitive advantage as key internal determinants [10]. The classic approach of a cleaner manufacturing strategy enhances organizational green competitive advantage through environmental preservation. Global organizations like the United Nations Division for Sustainable Development (UNDSD) and the International Federation of Accountants (IFAC) have promoted the use of environmental management accounting as a control system for implementing environmental strategies [11]. The field of strategic management places significant attention on firm-level strategy, control systems, and actions influenced by individual-level awareness [10]. Nevertheless, these links are still overlooked in academic studies. Perceived benefit (PB) is a component of managerial discretion according to upper echelon theory, as proposed by Hambrick and Finkelstein [12] in 1987. However, the influence of PB on the selection of a cleaner production strategy and environmental management accounting implementation has not been explored in the field of management accounting.

Upper echelon theory was initially developed by Hambrick and Mason [10] in 1984 [10] and further expanded upon by Hambrick et al., Hiebl [13, 14]. Recently, this theory has been sporadically applied in the realm of management accounting [8, 14–16]. This study investigates how CEOs’ attitude toward the environmental, influenced by customer pressure, impact their decisions on implementing cleaner production strategies, implementing environmental management accounting, and achieving green competitive advantage for their organizations. Additionally, the moderating role of PB is also taken into account.

Under customer pressure, a company’s green competitive advantage is determined by a combination of informal social conditions created by organizational leaders, strategic decisions, and formal standards such as an environmental management accounting system. Top executives in Vietnamese manufacturing enterprises should carefully combine these resources to obtain optimal green competitive advantage, so generating a long-term competitive edge for the enterprise. Furthermore, establishing the ideal green competitive advantage is a difficult challenge. It should be viewed as an opportunity to reform and make a positive impact. This study’s primary focus is on perceptions and behaviors of CEOs. This study is the first to examine the correlation among CEOs’ attitude toward the environmental, the choice of cleaner production strategy, the implementation of environmental management accounting, and green competitive advantage within a single research model. This study examines how CEOs’ environmental attitudes influence their decision-making strategies, the implementation of environmental management accounting, and gain the green competitive advantage in Vietnam’s transitioning economy. The PB ’s moderator role is considered in relation to two connections: (1) the CEO’s attitude toward the environmental and the choice of a cleaner production strategy, and (2) the CEO’s attitude toward the environmental and environmental management accounting implementation.

The study is structured as follows: it begins with an introduction, followed by a literature review and hypothesis development. The third section covers the research method, while the fourth section presents the results. The last parts consist of the conclusion, implications and limitations.

## 2. Literature review and hypothesis development

### 2.1. Upper echelon theory (UET)

**Fig 1:**
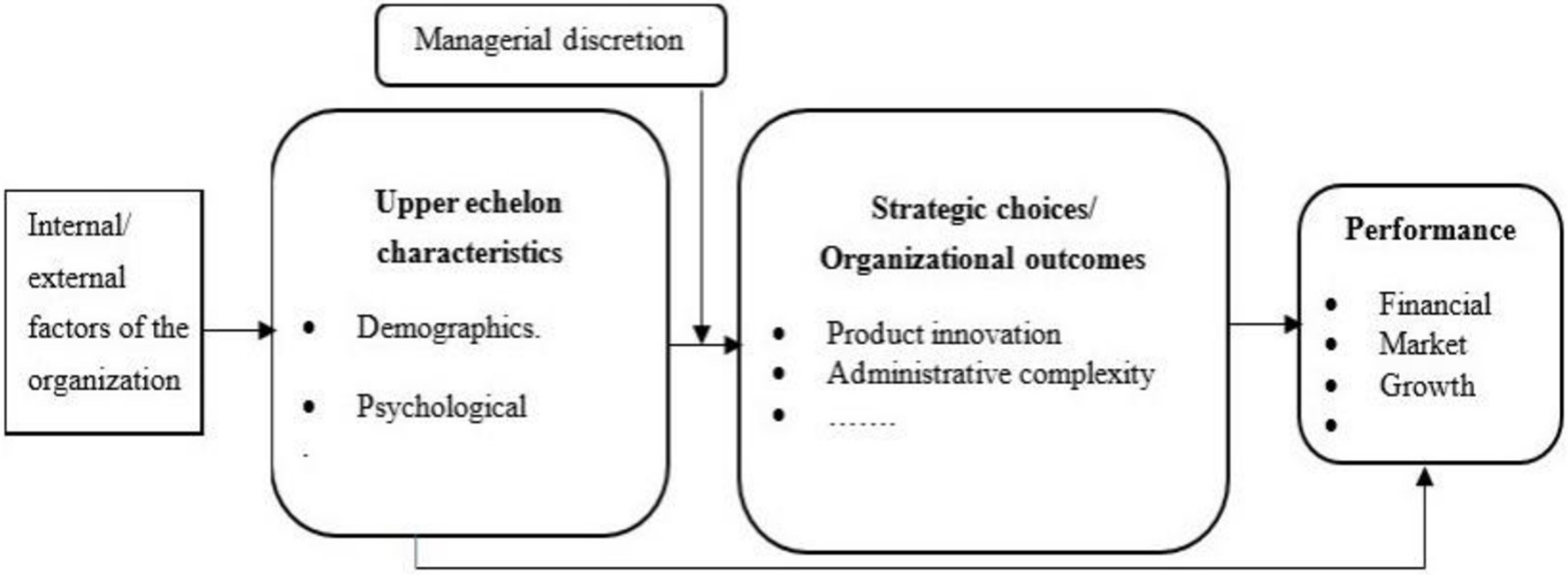
Conceptual model of UET. (Source: Adjusted from Hambrick & Mason (1984, p. 198) and Hiebl (2014, p. 225)).

Hambrick and Mason [10, 17] created a theoretical framework centered on the characteristics of top managers that deals with how organizations assess the business environment, make strategic decisions, and achieve performance. Fig 1 depicts the upper management’s perspective of organizations, which, according to UET, can help in comprehending the connections among internal and external factors of the organization, top managers’ attitudes, environmental strategy, complexity of the administration system, and organizational performance. This study highlighted the perceived benefit of cleaner production strategy and perceived benefit of environmental management accounting implementation as factors of management choice that would operate as moderator variables in the research model.

### 2.2. Customer pressure (CuP)

Consumers select products by evaluating which combination of product features best fulfills their requirements in terms of value, cost, and previous satisfaction [18]. Customers are valuable intangible assets of a company that need to be appreciated and effectively handled [19]. Ateş et al. [20] characterized customer pressure as the demands and expectations of end consumers and business clients for the company to diminish its impact on the environment. Ateş et al. [20] mentioned that customers exert non-regulatory pressure on many companies for environmental management, demanding that manufacturing firms minimize the negative impact of their products and operations on the natural environment. In recent decades, increasing environmental challenges and consumer consciousness have led to the development of several proposals for addressing sustainability issues in industrial production [21, 22].

### 2.3. Attitude toward the environmental (ATE)

Two concepts of psychological factors are personal values and attitudes. In previous literature, two types of environmental attitudes were identified: “(1) attitude towards the environment, and (2) attitude towards ecological behavior” [23]. The term ATE is reviewed along with personal values in this study. In certain ways, ATE reflect human predispositions that influence their behavior [24]. Based on the research of Schultz et al. [25], ATE is defined as “the collection of beliefs, affect, and behavior intentions a person holds regarding environmentally related activities or issues”. ATE concerns commonly refers to environmental concern [26], which some authors define as an affective environmental attitude. ATE is used interchangeably with environmental concern [27]. It refers to general concerns about the natural environment [23]. Among numerous research measures of attitude toward the environmental, the New Environmental Paradigm (NEP) scale, which was constructed by Dunlap et al. [28], is most popular because it evaluates general ATE rather than attitudes toward specific ecological behavior or some aspects of the environment [29]. NEP was developed with emerging economic awareness, challenging anthropocentric beliefs prevalent in Western society in the mid-1970s. The instrument has been used to assess individuals’ environmental concerns and generalized beliefs about human–environment relationships [28]. In reality, in the Vietnamese market today, many CEOs believe that consumers pay a lot of attention to green production factors and clean products that do not harm the environment and protect health. Therefore, CEOs consider customer demand for green and clean products to be the top priority in products manufactured today in Vietnam [6].

### 2.4. Cleaner production (CP)

Cleaner production is a proactive approach aimed at reducing the environmental impact of production and products, as stated by da Silva et al. [30]. Typically, this is defined as delivering quantifiable enhancements during the whole lifespan of the product [31]. This could be a result of a technological or procedural change in the development of a "cleaner" alternative. The product or its components may be recyclable, biodegradable, engineered for reuse, remanufacture, repair, or disposability. The sustainability of a product may be seen in various aspects, such as the materials used, waste production, product usage, disposal methods, pollution levels, and health and safety protocols [32]. Firms can strengthen their competitive advantage and corporate image by inventing goods, processes, and technologies that conserve energy, reduce pollution, recycle trash, or contribute to environmental management, a concept known as green differentiation [33]. Cleaner production is an effective technique that has shown positive outcomes in reducing environmental harm and generating economic and social advantages since its inception [34]. Customers are willing to pay more for products that have superior advantages such as traceability, quality standards, friendliness (green label), etc. [35]. The demand for green production processes and clean products is not only made by customers in Europe, Japan, the United States, etc., but also by consumers in the domestic market in Vietnam. The demand for healthy, green and clean products with little impact on the environment is one of the highlights today in Vietnam. Therefore, in order to increase competitiveness in the market and continue to operate and grow, Vietnamese manufacturers have no other way to follow the path of greener and cleaner production [6].

### 2.5. Environmental management accounting (EMA)

Bresciani et al. (2023, p.286) [36] defined EMA as the process of translating events and developing shared understanding and conceptual schemes among members of upper management, which explains the organizational processes and helps understand environmental information. For CEOs, EMA will be a useful system that can support them and assist them in reducing their company’s environmental impacts and making better decisions in the face of external pressure [37]. Information from EMA can assist CEOs to recognize, assemble, utilize, and analyze different kinds of accounting information for making informed and beneficial decisions regarding their company’s environmental management [38]. In essence, EMA and economic accounting data are aggregated together, which reflects the interrelationship between these two areas and corporate performance from managers’ decisions [39]. Organizations can trace, collect, collate and analyze environmental information from a system like EMA [40]. Not only EMA provide relevant information for management regarding the reduction of pollution, but it also support decision-making and performance management [40, 41].

### 2.6. Green competitive advantage (GCA)

Competitive advantage is identified by the differentiated position of the organization compared to competitors gained through resource exploitation [42]. In this study, to enhance the achievement of sustainable development, GCA is defined as a critical factor to consider. An organization will have a GCA when it implements a unique strategy that competitors cannot currently or potentially achieve or that they could generate similar benefits from [43]. Therefore, the organization’s GCA will give it a relative position in an industry [44]. An organization can also gain a GCA when it creates unique products that are accepted and appreciated by customers and cannot be imitated by competitors [45].

### 2.7. Perceived benefit

#### 2.7.1. Perceived benefit of cleaner production (PB_CP)

Multiple authors emphasize the significance of ISO14000 as a crucial instrument for facilitating cleaner production development and achieving advantages like new business prospects, enhancing organizational image, and strengthening relationships with stakeholders [46, 47]. The competitive industry climate and stakeholder pressures have necessitated environmental changes, resulting in various benefits for the business [48]. Severo et al. (2017) [47] found a significant association between cleaner production and product developments that result in favorable financial outcomes for companies. Bai et al. [49] show that mandatory audits of corporate performance for industrial firms have enhanced resource efficiency and reduced pollution. This has also influenced production organizations’ views on goods and the environment [50].

#### 2.7.2. Perceived benefit of EMA implementation (PB_EMA)

The main motivator for implementing environmental management methods is the perceived benefit [51]. Previous studies have consistently highlighted economic gain as a key motivator for using EMA [52]. One of the primary obstacles preventing enterprises from implementing EMA is the lack of information regarding its benefits [53]. Simpson et al. (2004) [54] suggested that small and medium-sized companies can also achieve substantial competitive benefits by adopting EMA. Many companies are hesitant to make the required investment and adopt EMA due to their perception that the resulting benefits are minimal [36]. If they enhance the perceived benefits, individuals are more likely to implement EMA [55]. Implementing EMA can provide organizations with cost savings, risk reduction, improved environmental performance, and increased competitive advantages [55, 56].

### 2.8. Hypothesis development

#### 2.8.1. The impact of customer pressure on choosing a cleaner production strategy

Consumers usually apply normative pressure in the manufacturing setting [57]. Customers are willing to compromise on functional performance to purchase a product that provides environmental advantages [58]. Progressive corporations began implementing proactive environmental strategies to suit customer demands rather than simply adhering to laws and regulations [20]. Not adhering to customers’ environmental preferences in a competitive market can result in a decrease in market share and impact financial performance, as stated by Gualandris & Kalchschmidt [59]. Cleaner production is increasingly favored due to its strong dedication to emission reduction, ability to respond positively to customer demands, and ease of obtaining recognition.

*H*_*1*_: *Customer pressure has a positive impact on choosing the cleaner production strategy*

#### 2.8.2. The impact of customer pressure on CEOs’s attitude toward the environmental

Research in industrialized nations indicates that customer demands have a significant role in influencing senior management to prioritize corporate social and environmental responsibilities [60]. Customers are identified as the primary stakeholders within organizations who raise awareness among hotel owners and senior management to implement environmentally friendly practices due to growing customer concerns about environmental degradation [61]. The managers acknowledged that consumers’ impressions of environmental management stems would positively influence managers’ support for environmental management practices [2]. Upper managers exhibit a positive ATE protection due to client demand, irrespective of their awareness of environmental activities. They acknowledge that environmental care holds greater significance in today’s market.

*H*_*2*_: *Customer pressure has a positive impact on CEOs’s attitude toward the environmental*

#### 2.8.3. The impact of customer pressure on environmental management accounting implementation

Initial research in organizational design emphasized the impact of the external environment on organizational structures [62]. Bohdanowicz [61] determined that consumer demands were the second most significant motivator for upper managers in European businesses to implement environmental friendly practices. EMA is a valuable instrument that can assist the firm in identifying, collecting, using, and evaluating various types of accounting information to make well-informed decisions regarding its environmental management. Organizations utilizing EMA can realize benefits such as enhanced business image and improved customer interactions [63]. In addition, the implementation of the EMA system is aimed at conforming to regulations, professional standards, and other stakeholders such as customers [63]. Lisi [64] emphasizes the important of the EMA system in aligning organizational behavior with customer demands.

*H*_*3*_: *Customer pressure has a positive impact on CEOs’s environmental management accounting implementation*

#### 2.8.4. The impact of CEOs’s attitude toward the environmental on cleaner production

Top management’s views on environmental issues impact the possibilities for implementing voluntary environmental strategies [65]. Companies led by top executives with a favorable environmental attitude are likely to prioritize natural environment concerns [66]. The owner-manager’s good attitude towards the natural environment is essential for developing and maintaining the firm’s proactive environmental strategy. Organizations’ top management plays a crucial role in implementing cleaner production practices. Organizations have gained economic and environmental advantages by implementing cleaner production methods [67]. The goal of a cleaner production strategy is to minimize the adverse effects of industrial activities on the environment while also meeting economic objectives [68].

*H*_*4*_: *The attitude toward the environmental of CEOs will have a positive impact on choosing a cleaner production strategy*.

#### 2.8.5. The impact of CEOs’s attitude toward the environmental on green competitive advantage

Core competencies encompass distinctive activities or goods that rivals find challenging to replicate [43]. The correlation between ATE and other factors, such as control systems like EMA adoption, plays a role in an organization’s long-term success [10]. A link between ATE and GCA is projected to exist [10]. A unique value strategy can be developed by upper management that is difficult for competitors to replicate, resulting in strategic benefits that are unmatched, and a positive attitude will lead top managers to select an environmentally sustainable business strategy or product, which will lay the groundwork for attaining a GCA [43]. The key factor for a company to achieve a GCA is the mentality of top management [43].

*H*_*5*_: *The attitude toward the environmental of CEOs will have a positive impact on green competitive advantage*.

#### 2.8.6. The impact of the CEOs’s attitude toward the environmental on environmental management accounting implementation

Schaltegger et al. [69] empirically demonstrated that various managers are interested in and handle diverse types of environmental information. Labodová [70] suggested that EMA must be incorporated into a comprehensive management system in order to be a valuable component of business administration. Executives who have a good attitude towards adopting environmental practices can drive their firms to become more involved in environmental management [71]. EMA is a tool used to monitor and uncover environment-related costs that are typically concealed within overhead expenses. It offers managers the necessary data to pinpoint opportunities within their companies by precisely computing and reallocating costs to specific products and processes. This enables the identification of inefficient processes with significant environmental consequences [72]. Senior executives recognize the advantages of implementing EMA as a waste management strategy, which can lead to economic gains for paper processing companies [72].

*H*_*6*_: *CEOs’ attitude toward the environmental will have a positive impact on environmental management accounting implementation*.

#### 2.8.7. The impact of cleaner production on green competitive advantage

Armenti et al. [73] emphasize the benefits of using cleaner production and pollution prevention methods compared to conventional end-of-pipe strategies. Furthermore, we saw that external market forces have influenced how smaller environmental enterprises gain access to consumer markets by requiring them to reveal certain actions and outcomes, as noted by Hicks & Dietmar [74]. The research portrays cleaner production as a helpful strategy for enhancing the business image and improving relationships with stakeholders [75]. Improvements in material utilization, productivity, cost reduction in processes, and pollution treatment have the potential to significantly reduce costs and enhance the profitability of an operation [76]. Researchers suggest that initiatives like cleaner production can aid in the development of countries and improve the long-term competitiveness of firms [77].

*H*_*7*_: *Cleaner production has a positive impact on green competitive advantage*.

#### 2.8.8. The impact of cleaner production on environmental management accounting implementation

For a good result, environmental initiatives need to be progressively included in various aspects of the overall organizational control system [78]. For a strategy to be successful, it must align with the organization’s structure and management control system [79]. EMA is viewed as a supportive tool for senior managers during the implementation of a cleaner production strategy and is likely to attract the attention of numerous experts. The utility of the EMA extends beyond a financial management perspective. Several South African organizations have found significant savings through effective environmental management by adopting Environmental Management Accounting (EMA) to precisely track and recognize environmental expenses [80]. Schaltegger et al. (2010, pp.17-19) [81] employed EMA, cleaner production evaluation, and environmental management systems to evaluate organizations’ sustainable performance. The research provided favorable outcomes and enhanced cleaner production assessment initiatives by highlighting the financial consequences of EMA.

*H*_*8*_: *The cleaner production strategy will have a positive impact on EMA implementation*.

#### 2.8.9. The impact of environmental management accounting implementation on green competitive advantage

Managers have recognized that the greatest benefit provided by EMA is its usefulness in helping to discover opportunities to improve corporate reputation and the long-term decision making of the organization [82]. Specifically, implementing EMA helps businesses improve their environmental performance and save costs, thereby helping to improve pricing decisions, reduce resource waste, and increase revenue, while market access is also promoted, improving the efficiency of investment capital [52, 83]. EMA provides a platform for managers to recognize the tension between economic growth and adverse environmental impacts, and to identify actions to reduce environmental impact while improving economic performance [40]. EMA can also be considered an intangible benefit. Masanet-Llodra [84] offers evidence that firms try to gain competitive advantages through innovation using EMA systems.

*H*_*9*_: *The implementation of environmental management accounting will have a positive impact on green competitive advantage*.

#### 2.8.10. The moderator role of perceived benefit

The main motivator for implementing an environmental strategy is the perceived advantage and it is useful for examining the connection between CEOs’ ATE issues and the selection of cleaner production strategy [51]. Cobra et al. [85] mentioned that the main benefit of cleaner production is the reduction in waste produced during the manufacturing process, which could otherwise result in waste and spoilage. Cleaner production application is mostly observed in energy efficiency, water preservation, and minimizing the use of materials and resources [86]. Yüksel [87] shows that managers who adopted cleaner production saw a beneficial effect on how their products and services were perceived by linking them to the company’s environmental efforts, which might improve the organization’s image and cultivate better customer connections. Organizational competitiveness is frequently analyzed on its own due to its combination of tangible and intangible attributes that impact profitability, as stated in earlier studies [88].

*H*_*10a*_: *When perceived benefits of cleaner production are higher by CEOs*, *the impact of CEOs’ attitude toward the environmental on choosing cleaner production strategy will be stronger*.

Environmental management practices are primarily motivated by the perception of benefit; therefore, it is useful to comprehend the connections between CEOs’ ATE and the implementation of EMA [51]. Economic benefit has been identified as a significant motivator for EMA implementation in the majority of prior literature, and as far as we are aware, benefit perception motivates organizations to implement EMA [52]. One of the primary obstacles impeding firms from implementing EMA is the need to provide information regarding its benefits [53]. There is a high probability that senior executives will implement EMA if the perceived benefits are enhanced in some fashion. Indeed, the adoption of EMA can yield numerous advantages for organizations, including cost savings, mitigation of environmental and social hazards, enhancement of environmental performance, and bolstering of competitive advantages [56, 83]. Therefore, in light of the impact that CEOs’ attitudes have on the environment, companies that perceive substantial advantages will have a greater propensity to adopt EMA.

*H*_*10b*_: *When perceived benefits of environmental management accounting implementation are higher by CEOs*, *the impact of CEOs’ attitude toward the environmental on environmental management accounting implementation will be stronger*.

**Fig 2:**
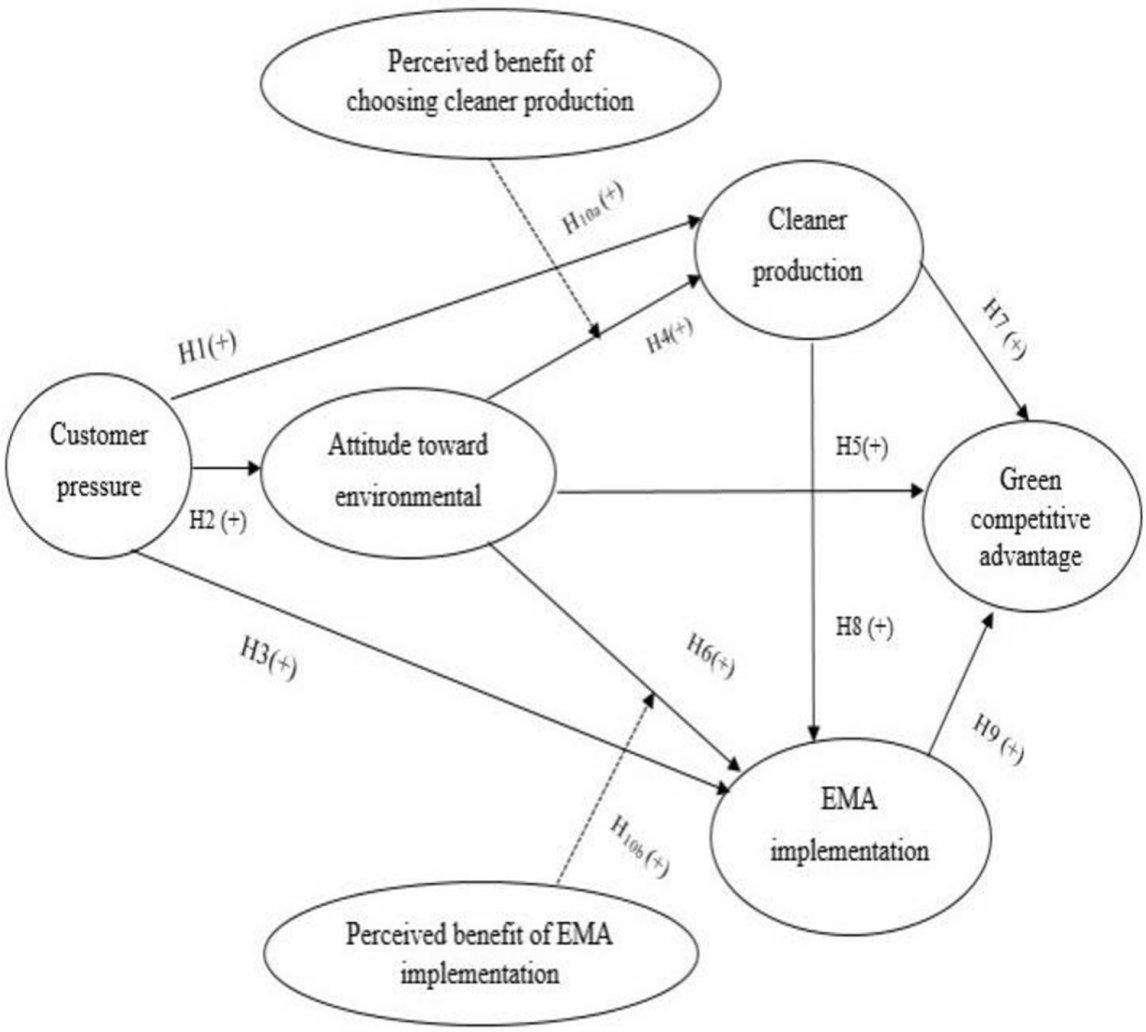
Research model. Source: Own developed.

The proposed research model is presented in Fig 2.

## 3. Research methodology

### 3.1. Research design

The type of study is descriptive-explanatory. Several research types could classify this investigation as descriptive-explanatory in nature, given that its objectives are to provide descriptions of specific events or situations and to elucidate the interconnections among variables. The research philosophy is based on positivism. A philosophy similar to positivism influences this study, emphasizing the development of hypotheses based on established theories. Subsequent to testing and confirming or rejecting these hypotheses, additional research is conducted to refine the theories [89]. Using highly structured methods to gather quantitative data (e.g., questionnaires), management research continues to favor positivism.

A deductive approach was used to this research. Consistent with the positivist paradigm, the present study employs a deductive methodology. As stated by Saunders et al. [89], this methodology is distinguished by the following: (1) It employs highly structured methods to establish and test hypotheses regarding causal relationships between variables; (2) It operationalizes concepts to enable the quantitative measurement of facts; and (3) It selects a sufficient number of samples to statistically generalize about regularities in human social behavior. Research method: mixed methods. This study collects data through utilization questionnaires. The quantitative and qualitative data are subsequently analyzed quantitatively and qualitatively, respectively. By adopting this methodology, one can effectively harness the benefits of qualitative and quantitative approaches [90].

### 3.2. Variables measurement, sampling strategy and sample collection

#### Variables measurement

This study inherited the scales from previous studies. Customer pressure was studied by Chu et al. [91], comprising four items. The attitude toward the environmental scale consists of 15 items, based on research by Dunlap et al. (2000) [28]. The cleaner production scale was inherited research by Severo et al. [92] and consists of five items. The EMA scale is based on a study by Chaudhry et al. [63], which consists of six items. The GCA scale consists of four items and is based on research by Lin and Chen [93]. The PB_CP scale consists of nine items, based on research by Matos et al. [75]. The scale of PB_EMA implementation is based on research by Wang et al. [94]. All items used a 5-point Likert scale, of which 1 = strongly disagree and 5 = strongly agree.

#### Sampling strategy and sample collection

As it stands, most studies on EMA have been done on manufacturing companies because their actions are harmful for the environment and they use a lot of natural resources [95]. In this study, the manufacturing companies were also used as the unit of analysis. The way CEOs think, and act is a big part of strategic planning, how executives act when they use accounting data to make choices. CEOs of manufacturing companies have a big impact on both the strategic decisions that are made and the actions that are taken to carry out those decisions. In this case, the people who answered the poll were CEOs of companies. The southern part of Vietnam has the most manufacturing firms and the most manufacturing firms overall [96]. This is because the southern part of Vietnam is geographically more favorable and has better economic conditions. Water and air pollution are getting worse in this area, and other natural problems are getting worse too. So, studying how the EMA is used in this area is interesting, important, and could have effects on businesses and governments in Vietnam.

As part of the study, respondents were chosen conveniently by two methods: (1) the questionnaire was sent directly to respondents through the authors’ contacts, and (2) respondents were sent a questionnaire via Google form. With the first method, researchers chose 500 CEOs to fill out the questionnaire based on the relationships we have with them. With the second method, the study team used a list of information provided by government agencies to pick 2,500 CEOs of manufacturing firms at random. Staff from local government agencies helped us do this.

Seven hundred and twenty-four businesses decided to take part in this survey. With two different approaches, the study team sent each company one questionnaire and one instruction on how to fill it out. This was done to reduce the effect of single-respondent bias and improve the accuracy of the data. The instructions for filling out the form also reassured CEOs that their answers would remain anonymous and private and would never be shared with anyone else. To get more CEOs to respond, follow-up calls and emails were sent to remember them, and companies were told they could get the data analysis report if they needed to. In total, 415 CEOs filled out the survey and sent back the questionnaires. The people on the study team called the people who didn’t answer the survey. They said they didn’t have time to finish the questionnaires because they felt they were too personal and touched on sensitive topics like the environment and money. The study team carefully looked over the questionnaires and threw away the ones that were missing data, incomplete, or from small manufacturers. In the end, 234 useful surveys are sent back, which is 7.8% of the total 3,000. Table 1 shows more information about the companies that were chosen for the group.

**Table 1:** Demographics of the participating firms and respondents.

Tenure	Freq.	%	Gender	Freq.	%
1–5 years	32.0	14.0	Male	150.0	64.0
6–10 years	80.0	34.0	Female	84.0	36.0
11–15 years	51.0	22.0	**Total**	**234.0**	**100.0**
15–20 years	56.0	24.0	**CEO’s Education**	Freq.	%
>20 years	14.0	6.0	Pre-undergraduates	5.0	2.1
**Total**	**234.0**	**100.0**	Graduates	87.0	37.2
**Type of manufacturing**	**Freq.**	**%**	Post-graduates	142.0	60.7
Textiles, leather and shoes	92	39.3	**Total**	**234.0**	**100.0**
Plastic, packaging	44	18.8	**Founded time of firms**	**Freq.**	**%**
Mechanical machines	12	5.1	< 5 years	12.0	5.1
Pharmaceutical	23	9.8	6–10 years	105.0	44.9
Process the wood	28	12.0	11–25 years	80.0	34.2
Food production	9	3.9	> 25 years	37.0	15.8
Other production	26	11.1	**Total**	**234.0**	**100.0**
**Total**	**234.0**	**100.0**	**Questionnaires**	**Freq.**	
**CEO’s age**	**Freq.**	**%**	Sent	3,000	
18–29	19.0	8.1	Received	415.0	
30–49	98.0	41.9	Missing value	7.0	
50–64	91.0	38.9	Excluding small firm	174.0	
>64	26	11.1	Final sample	234.0	

### 3.3. Estimation techniques

To obtain official results, we employed the SmartPLS3.3.0 program, which performs two PLS-SEM model evaluation processes: (1) measurement model assessment and (2) structural model assessment. Measurement model assessment includes a range of assessments: (1) Common method bias test, internal consistency reliability assessment (2) Convergent validity assessment, taking into account the reliability of each measuring scale (outer loading) and average variance extracted (AVE), and (3) discriminant validity assessment using additional Fornell-Larker criteria as well as HTMT criteria. Structural model assessments include multi-collinearity problem assessment, path coefficient assessments, predictive relevance (Q^2^), coefficient of determination (R^2^), and effect size (f^2^).

## 4. Results

For fundamental statistical analysis, SPSS 24 is employed. Path analysis is the main application of partial least squares structural equation modeling (PLS-SEM) [97]. This section presents the results of the empirical analysis, including the reliability and validity of the model, testing of hypotheses.

### 4.1. Measurement model

Harman’s single-factor test was used to assess the issue of common method bias. All measurement constructs were subjected to an un-rotated factor analysis in order to identify seven components. The combined variance of the seven components is 68.5%. The first element accounts for only 40.56% of the total. As a result, the overall cumulative variance errors of the entire model (50%) are not a significant issue in this study [98].

The model’s reliability was assessed using Cronbach’s alpha, with values over 0.7 deemed acceptable, and composite reliability, with values equal to or more than 0.6 as regarded acceptable [99]. The Cronbach’s alpha and composite reliability indexes in Table 2 are significantly higher than the recommended values, demonstrating the variables’ reliability. Validity mostly pertains to convergent and discriminant validity.

**Table 2:** Construct reliability and validity.

Research constructs/Items	Outer Loadings	Α	rhoA	C.R	AVE
**Attitude toward the environmental** [28]					
**ATE1:** The Earth’s carrying capacity is nearing its maximum.	0.763	0.949	0.950	0.954	0.583
**ATE2:** Humans possess the prerogative to alter the natural environment in order to accommodate their own requirements.	0.788				
**ATE3:** Human intervention in nature frequently results in catastrophic outcomes.	0.797				
**ATE4:** Human resourcefulness will guarantee that we are not making the planet uninhabitable.	0.791				
**ATE5**: Humans are grossly exploiting the environment.	0.771				
**ATE6**: If we simply learn how to exploit the ample natural resources available, they will continue to do so.	0.735				
**ATE7**: Plants and animals possess an equal right to existence alongside humans.	0.734				
**ATE8**: The equilibrium of nature is robust enough to withstand the effects of contemporary industrialized nations.	0.751				
**ATE**9: Notwithstanding our unique capabilities, human beings remain vulnerable to the forces of nature.	0.738				
**ATE10**: The purported "ecological crisis" that confronts humanity has been grossly inflated.	0.757				
**ATE11:** The Earth is a spacecraft with extremely limited resources and space.	0.761				
**ATE12**: Humans were designed to dominate the remainder of nature	0.791				
**ATE13**: The equilibrium of nature is exceedingly precarious and prone to disruption.	0.783				
**ATE14**: Over time, humanity will acquire sufficient knowledge regarding the mechanisms of nature to exert control over them.	0.724				
**ATE15**: We will shortly be confronted with a catastrophic ecological event if current trends continue.	0.799				
**Cleaner production** [92]					
**CP1:** As a result of cleaner production, refuse emissions were reduced.	0.663	0.838	0.843	0.887	0.612
**CP2:** Raw material consumption was reduced as a result of cleaner production.	0.724				
**CP3:** Energy consumption was reduced due to cleaner production.	0.860				
**CP4:** Water usage was reduced due to cleaner production.	0.804				
**CP5:** cleaner production contributed to a reduction in environmental impact.	0.843				
**Customer pressure** [91]					
**CuP1:** The organization observes the impact of consumers’ environmental concerns.	0.680	0.781	0.796	0.858	0.604
**CuP2**: The organization is under pressure to establish a green reputation.	0.852				
**CuP3:** The company is under Customer pressure regarding environmentally friendly packaging.	0.809				
**CuP4**: Customer contracts will be terminated if the company fails to comply with their environmental requirements	0.758				
Environmental management accounting [63]					
**EMA1:** The accounting system utilized by our organization to record every physical input and output (including energy, water, materials, waste, and emissions).	0.780	0.863	0.868	0.898	0.595
**EMA2:** The accounting system of our organization is capable of conducting analyses on product inventories, product enhancements, and product environmental impacts.	0.742				
**EMA3:** Our organization employs environmental performance targets (EMA3) for both tangible inputs and outputs.	0.849				
**EMA4**: Environmental costs and liabilities can be identified, estimated, and categorized by our company’s accounting system.	0.789				
**EMA5:** The accounting system of our company is capable of generating and utilizing environmental-related cost accounts	0.749				
**EMA6**: The accounting system of our organization has the capability to assign environmental-related expenses to individual products and environmental-related costs to products.	0.711				
**Green competitive advantage** [93]					
**GCA1**: In comparison to its market leaders, the organization possesses a competitive advantage in the form of cost-effective green innovation and environmental management.	0.912	0.920	0.920	0.943	0.806
**GCA2**: The company provides green products or services of a higher quality than its principal competitor.	0.911				
**GCA3**: Green innovation and environmental R&D capabilities surpass those of the company’s main competitors.	0.889				
**GCA4**: Environmental management capabilities are superior to those of the company’s main competitors	0.878				
**Perceived benefit of Cleaner production** [75]					
**PB_CP1**: Reduction of pollution, waste, and GHG emissions.	0.867	0.813	0.824	0.842	0.588
**PB_CP2**: Process, productivity, and product efficiency improvements (energy, water, materials, and use and reuse of productive resources)	0.822				
**PB_CP3**: Reduction of risks (occupational, human, and environmental)	0.712				
**PB_CP4**: New business opportunities (market access and innovation in sustainable products and processes)	0.825				
**PB_CP5**: Improvement of the organizational image and strengthening of the relationship with stakeholders.	0.852				
**PB_CP6**:Organizational competitiveness and profitability	0.713				
**PB_CP7**: Improvement of the work environment (environment, workers and managers qualifications, motivation)	0.770				
**PB_CP8**: Quality and improvement of product safety for consumers	0.745				
**PB_CP9:** Technological update of productive processes	0.896				
**Perceived benefit of EMA implementation** [94]					
**PB_EMA1**: The implementation of environmental management accounting will enhance the legitimacy and competitiveness of your organization.	0.876	0.842	0.853	0.875	0.604
**PB_EMA2**: The implementation of environmental management accounting is advantageous for mitigating the environmental expenses and consequences of your organization, thereby bolstering its reputation.	0.859				
**PB_EMA3**: Environmental management accounting facilitates the reduction of operational expenses and the identification of new opportunities for your organization.	0.797				
**PB_EMA4**: The integration of environmental management accounting can furnish our organization with diverse data to facilitate informed decision-making and enhance overall performance.	0.890				

Convergent validity was evaluated using standardized loadings and average variance extracted (AVE) as proposed by [99]. All loadings in Table 2 exceed the recommended minimum value of 0.5. Each construct’s average variance extracted (AVE) exceeds the required threshold of 0.5. Overall, the results offer broad confirmation of convergent validity among the constructs.

The discriminant validity of the five components was assessed by comparing the square root of the average variance extracted (AVE) with the potential inter-construct correlation coefficient [99]. According to the Fornell-Larcker criterion results in Table 3, the square root of the AVE was consistently greater than the correlation between all pairs of constructs, indicating acceptable discriminant validity.

**Table 3:** Fornell-Larcker criterion.

	ATE	CP	CuP	EMA	GCA
ATE	0.764				
CP	0.762	0.782			
CuP	0.555	0.631	0.777		
EMA	0.689	0.718	0.745	0.771	
GCA	0.789	0.735	0.533	0.700	0.898

In addition, the results of 2,000 bootstrap runs reveal that value 1 is not included in the confidence interval of HTMT values from 2.5% to 97.5% (Table 4). The measurement scale therefore attains the discriminant value.

**Table 4:** Heterotrait-Monotrait Ratio (HTMT).

Constructs	ATE	CP	CuP	EMA	GCA
ATE					
CP	0.851				
CuP	0.638	0.775			
EMA	0.759	0.849	0.898		
GCA	0.836	0.837	0.634	0.778	

### 4.2. Structural model

According to Hair et al. (2017) [100], the variance-inflating factor (VIF) is used to assess multi-collinearity between independent variables. The research model is divided into three models, each with one dependent variable, due to the large number of dependent variables. The remaining scales do not seem to exhibit multi-collinearity because their VIF is less than two.

**Fig 3:**
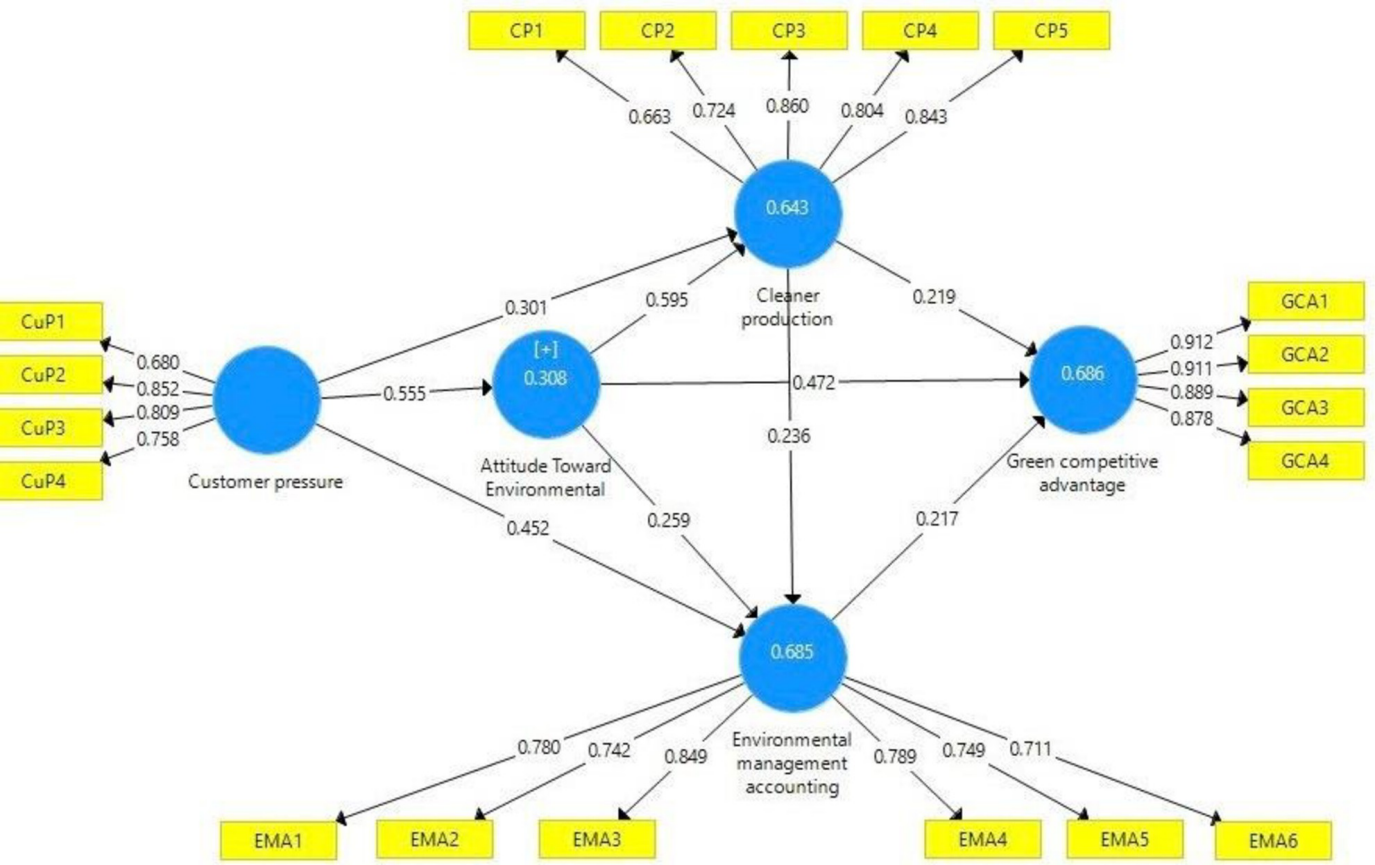
PLS-SEM analysis results of the theoretical model.

The ability of the independent variables to predict outcomes is commonly measured using the coefficient of determination (R^2^). Results in Fig 3 indicate that ATE has a weak R^2^ value (0.308); cleaner production has a R^2^ value that may be considered to have a high degree of prediction (0.643); EMA variable has a strong R^2^ value (0.685) and the GCA variable has the strongest R^2^ value (0.686) [100].

The out-of-sample predictive power is also assessed using predictive relevance (Q^2^). The results of Table 5 further demonstrate that the dependent variables’ Q^2^ coefficients are greater than zero, indicating the model’s capacity for prediction [100]. The significant effect size (f^2^) is employed to assess R^2^. The term exclusion refers to the act of removing a certain independent variable from the study framework and then examining its subsequent impact on the dependent variable [100]. Hair et al., (2014) [101] suggest that in order to assess the magnitude of the impacts of independent variables, it is advisable to compare f^2^ values with thresholds of 0.02 (weak), 0.15 (moderate), and 0.35 (strong). Based on the results, it can be observed that ATE (f^2^ = 0.142) and CP (f^2^ = 0.148) exhibit small effects, however, EMA (f^2^ = 0.285) demonstrates a significant influence on green competitive advantage.

**Table 5:** Hypothesis testing.

Relationships	Original Sample (O)	Sample Mean (M)	Standard Deviation (STDEV)	Sig.	Results
H1: CuP-> CP	0.301	0.299	0.045	0.000	Supported
H2: CuP -> ATE	0.555	0.559	0.075	0.000	Supported
H3: CuP -> EMA	0.452	0.459	0.070	0.000	Supported
H4: ATE -> CP	0.595	0.597	0.037	0.000	Supported
H5: ATE -> GCA	0.472	0.476	0.084	0.000	Supported
H6: ATE -> EMA	0.259	0.252	0.091	0.004	Supported
H7: CP -> GCA	0.219	0.213	0.092	0.018	Supported
H8: CP -> EMA	0.236	0.236	0.069	0.001	Supported
H9: EMA-> GCA	0.217	0.217	0.075	0.004	Supported

Q^2^ Attitude toward the environmental: 0.116

Q^2^ Cleaner production: 0.227

Q^2^ Environmental management accounting: 0.302

Q^2^ Green competitive advantage: 0.413

PLS-SEM analysis was performed to examine the proposed hypotheses in our study (Fig 3). The results of the estimation of the structural equation model are presented in Table 5. As can be seen from Table 5, Customer pressure has a significant positive impact on both choosing cleaner production, ATE and EMA implementation (Sig.<0.05). Thus, both hypotheses H_1_, H_2_, H_3_ are fully supported. Also, ATE was found to be significantly and positively correlated with the cleaner production, GCA and EMA implementation (Sig. < 0.05), supporting hypotheses H_4_, H_5_ and H_6_. Cleaner production has a significant positive impact on GCA (Sig. = 0.018<0.05) as well as on EMA implementation (Sig. = 0.001<0.05). As a result, both hypotheses H_7_ and H_8_ are supported. There is also a positive and significant relationship between EMA implementation and GCA (Sig. = 0.004<0.05). Thus, hypothesis H_9_ is also supported.

One of the primary goals of this study is to examine the moderating role of PB_CP and PB_EMA implementation. PB_CP and PB_EMA implementation measurement concepts are reliable, according to analysis of the measurement model of moderating variables (Cronbach’s alpha > 0.8, Composite Reliability > 0.9, and AVE value > 0.7, square root 2 of two variables’s AVE value is greater than the correlation coefficient between the structures, and the confidence interval of the two variables does not contain a value of 1).

Next, the two-stage method proposed by Chin et al. [102] was applied to evaluate the influence of the moderating variable. While stage 1 estimated the primary effect model. Stage 2 multiplies the moderating and exogenous variables to evaluate the interaction (attitude toward the environmental x Perceived benefit of cleaner production; attitude toward the environmental x Perceived benefit of EMA).

The link between ATE and cleaner production strategy is moderated by CEOs’s PB_CP, as shown in Table 6 (β = 0.134; Sig. = 0.020). The link between ATE and EMA implementation is moderated by CEOs’s PB_EMA (β = 0.128, Sig. = 0.025). The impact of attitude toward the environmental on cleaner production strategy choice will be greater when the CEOs‘s PB_CP higher. The impact of attitude toward the environmental on EMA implementation will greater when the CEOs’s PB_EMA increase. Therefore, hypothesis H10a and H10b are supported.

**Table 6:** Testing results for moderator effects.

Relationships	Coefficient	R^2^	p–value
ATE -> CP	0.319	0.315	0.000
PB_CP -> CP	0.154		0.002
ATE x PB_CP -> CP	0.134		0.020
ATE -> EMA implementation	0.215	0.324	0.005
PB_EMA implementation -> EMA implementation	0.142		0.008
ATE x PB_EMA implementation -> EMA implementation	0.128		0.025

## 5. Conclusion

This study investigates the impact of customer pressure on a variety of relationships. The findings from the data analysis indicate that customer pressure has a statistically significant and positive impact on the selection of cleaner production and attitude toward the environmental, as well as the implementation of environmental management accounting. These results indicate that companies with a greater customer pressure are more likely to select the cleaner production strategy, attitude toward the environmental by CEOs, and implementation of environmental management accounting in order to establish and maintain positive customer relationships and legitimacy. The results of the regression analysis indicate that customer pressure has the most significant impact on attitude toward the environmental, suggesting that the customer may have a greatest influence on the CEO with attitude toward the environmental than on any other relationship that the customer affects. This phenomenon could potentially be attributed to the substantial influence that CEOs’ environmental behavior decisions are impacted by the critical role that customers play in environmental protection [94]. This result corroborated the findings of Bohdanowicz; Chaudhry et al. and Sloan et al. [2, 22, 61, 63].

Environmental attitudes have a direct influence on obtaining a green competitive advantage and a significant impact on decision-making regarding cleaner production strategies and environmental management accounting implementation. Among the three attitude toward the environmental-impact relationships, attitude toward the environmental and environmental management accounting implementation have the weakest correlation. The reason is that environmental management accounting implementation in Vietnam is still in its infancy; few businesses have effectively implemented environmental management accounting, and the majority of businesses are unsure of how to implement it in practice. The findings of this research further substantiate the correlation between the mindset of CEOs and the organizational control system, as postulated by Hambrick & Mason [10]. This is consistent with the hypotheses put forth by Labodová and Sharma [65, 70].

In addition, a direct and significant correlation was discovered between cleaner production strategy and green competitive advantage. It is determined that there is a significant correlation between cleaner production strategy and green competitive advantage; this is probably due to the minor magnitude of the effect. The findings indicate that cleaner production strategies may serve as a weapon by influencing the degree to which an organization undertakes efforts to gain green competitive advantage. This finding is consistent with the conclusions drawn by Kjaerheim [77] and Matos et al. [75], Sibarani & Genoveva [35]. Additionally, the study inquired about the significance of the cleaner production strategy in connection with environmental management accounting implementation. There was a suggestion that organizations adhering to a cleaner production strategy would have a higher propensity to implement environmental management accounting as a mechanism to accomplish their strategic goals. The results indicate that environmental management accounting implementation is influenced by strategy, which is consistent with the findings of Gosselin [103] that organizations are inclined to embrace management accounting innovations.

Moreover, this research revealed that the implementation of environmental management accounting influences green competitive advantage. The findings are completely persuasive due to the fact that the PLS structural model demonstrates a significant path. The correlation analysis result aligns with assertions that the utilization of environmental management accounting is probable to lead to the discovery of advantageous circumstances, including cost reductions and enhancements in manufacturing procedures [104, 105].

The perceived benefit of cleaner production strategies or perceived benefit of environmental management accounting positively influences the connection between attitude toward the environmental and the decision to choose a cleaner production strategy or implement environmental management accounting. These results indicate that the impact of attitude toward the environmental on selecting a cleaner production strategy or environmental management accounting implementation is heightened when the perceived benefits of cleaner production or environmental management accounting implementation are significant. Perceived benefits have a crucial role in organizations’ decisions to choose a cleaner production strategy or apply environmental management accounting. CEOs are more likely to endorse something when they see its benefits. With the CEOs’ support, implementing cleaner production or environmental management accounting would be easier and could secure necessary resources, including investments, employees, and techniques. Companies that highly value the cost-saving strategy or environmental management accounting implementation are more likely to have the capability and motivation to put them into practice. Therefore, enterprises that feel high benefits from the customer pressure are more inclined to adopt cleaner production and environmental management accounting to comply with the demand, ensuring positive connections and gaining legitimacy and repute. If not, their products may be rejected by customers, experience external resource depletion, and lose market share [106]. Thus, perceived benefits enhance these effects. Furthermore, the lack of uniform national and industry norms for the adoption of cleaner production and environmental management accounting leads companies with strong perceived benefits to be more susceptible to mimetic pressure, resulting in the implementation of cleaner production and EMA according to their own guidelines. The favorable influence of attitude toward the environmental on the execution of cleaner production and environmental management accounting relies on the perceived benefits of implementing cleaner production and environmental management accounting. This study’s findings supported the studies conducted by Alves & Oliveira [88] and Saeidi et al. [56], Cudjoe et al. [50].

## 6. Theoretical and practical implications

On the theoretical side, this is the first study that has attempted to develop a research model to measure the relationships between: external environment (Customer pressure)–personality of CEOs (attitude toward the environmental)–cleaner production (a strategy)—environmental management accounting implementation (Control system of organization)–green competitive advantage (Performance). Besides that, the relationships between the environmental attitude of CEOs and the choice of cleaner production strategy, combined with the implementation of the environmental management accounting aimed at a sustainable competitive advantage, are still not much of an interest in the world and in Vietnam. So this research contributes to filling this gap. In addition, this study is based on UET, a theory that focuses on the personality of upper managers in organizations, studying their behavior that will affect outputs, as well as the effectiveness of organizations through their strategic decisions. However, the use of UET in accounting is still not much of an interest for researchers.

On the practical side, first, considering the importance of customer pressure on attitude toward the environmental, cleaner production, implementing environmental management accounting by firm. Any company that makes a strategy that goes against consumer bias, its image will go down, thereby affecting its own market share. The results of this study confirm the importance of customers to the environmental-oriented production psychology of CEOs in Vietnamese manufacturing enterprises. Firms could hire professionals to provide training programs for top managers on implementing the cleaner production strategy and environmental management accounting, emphasizing the importance and potential benefits of these strategies. It is essential to promote awareness among top managers about organizations that have effectively implemented cleaner production and EMA, as well as the benefits they have gained. These measures are beneficial for enhancing the perceived benefits of cleaner production and environmental management accounting. The lack of industrial standards and procedures for implementing the cleaner production strategy and environmental management accounting could explain the minimal impact of Customer pressure. Therefore, professional organizations in strategy and accounting should offer more comprehensive guidance on cleaner production strategy and environmental management accounting implementation to meet the diverse needs of various companies. Industry groups should establish national and industrial standards for the implementation of the cleaner production strategy and environmental management accounting.

## 7. Limitations and directions for future research

This study has some limitations. To begin, it is important to note that this study exclusively examines two moderator variables: the perceived benefit of cleaner production strategies strategy and the perceived benefit of environmental management accounting implementation. Future research should concern other factors: environmental strategy, organizational coordination, and the comprehensiveness of an environmental management system, which are all crucial for businesses to engage in environmentally responsible conduct [107]. Additionally, further investigation in this area could examine the impacts of external pressures that modify the variables. Subsequent investigations may employ longitudinal designs to gather data in order to further examine the incidental relationships. Survey-based research is susceptible to response and social desirability biases as well. Subsequent investigations may contemplate augmenting the findings with objective data or employing a hybrid methodology combining a mixture of techniques, such as surveys and interviews, in order to bolster the conclusions drawn.

## Supporting information

S1 Data (CSV)

## References

[1] Chen C. and Ho H., “Who pays you to be green? How customers’ environmental practices affect the sales benefits of suppliers’ environmental practices,” *J*. *Oper*. *Manag*., vol. 65, no. 4, pp. 333–352, 2019.

[2] Sloan P., Legrand W., and Chen J. S., “Factors influencing german hoteliers’attitudes toward environmental management,” in *Advances in hospitality and leisure*, Emerald Group Publishing Limited, 2005, pp. 179–188.

[3] Yang M. G. M., Hong P., and Modi S. B., “Impact of lean manufacturing and environmental management on business performance: An empirical study of manufacturing firms,” *Int*. *J*. *Prod*. *Econ*., vol. 129, no. 2, pp. 251–261, 2011.

[4] Wyss A. M., Knoch D., and Berger S., “When and how pro-environmental attitudes turn into behavior: The role of costs, benefits, and self-control,” *J*. *Environ*. *Psychol*., vol. 79, p. 101748, 2022.

[5] Beresford M., “Doi Moi in review: The challenges of building market socialism in Vietnam,” *J*. *Contemp*. *Asia*, vol. 38, no. 2, pp. 221–243, 2008.

[6] Kinhte Saigon, “Manufacturer before pressure ‘greening’ or losing customers,” *KinhteSaigon March 26 (in Vietnamese)*, 2023. [Online]. Available: https://thesaigontimes.vn/nha-san-xuat-truoc-ap-luc-xanh-hoa-hoac-mat-khach-hang/.

[7] Nishitani K., Nguyen T. B. H., Trinh T. Q., Wu Q., and Kokubu K., “Are corporate environmental activities to meet sustainable development goals (SDGs) simply greenwashing? An empirical study of environmental management control systems in Vietnamese companies from the stakeholder management perspective,” *J*. *Environ*. *Manage*., vol. 296, p. 113364, 2021. 34399553 10.1016/j.jenvman.2021.113364

[8] Vo L. T., Van Vo N., Ngoc Pham T., and Hien N. N., “The Impact of Historical Performance and Managerial Risk-taking Propensity on the Behavior of Choosing Prospector Strategy and Using Strategic Management Accounting Information in Viet Nam Manufacturer,” *SAGE Open*, vol. 13, no. 4, p. 21582440231219360, 2023.

[9] Liem V. T., “The impact of education background on using cost management system information,” *Cogent Bus*. *Manag*., vol. 8, no. 1, p. 1944011, 2021.

[10] Hambrick D. C. and Mason P. A., “Upper echelons: The organization as a reflection of its top managers,” *Acad*. *Manag*. *Rev*., vol. 9, no. 2, pp. 193–206, 1984.

[11] Appannan J. S., Mohd Said R., Ong T. S., and Senik R., “Promoting sustainable development through strategies, environmental management accounting and environmental performance,” *Bus*. *Strateg*. *Environ*., vol. 32, no. 4, pp. 1914–1930, 2023.

[12] Hambrick D. C. and Finkelstein S., “Managerial discretion: A bridge between polar views of organizational outcomes.,” *Res*. *Organ*. *Behav*., 1987.

[13] Hambrick D. C., Finkelstein S., and Mooney A. C., “Executive job demands: New insights for explaining strategic decisions and leader behaviors,” *Acad*. *Manag*. *Rev*., vol. 30, no. 3, pp. 472–491, 2005.

[14] Hiebl M. R. W., “Upper echelons theory in management accounting and control research,” *J*. *Manag*. *Control*, vol. 24, no. 3, pp. 223–240, 2014.

[15] Liem V. T. and Hien N. N., “Exploring the impact of dynamic environment and CEO’s psychology characteristics on using management accounting system,” *Cogent Bus*. *Manag*., vol. 7, no. 1, 2020, doi: 10.1080/23311975.2020.1712768

[16] Zor U., Linder S., and Endenich C., “CEO characteristics and budgeting practices in emerging market SMEs,” *J*. *Small Bus*. *Manag*., vol. 57, no. 2, pp. 658–678, 2019.

[17] Hambrick D. C., “Upper echelons theory: An update.” Academy of Management Briarcliff Manor, NY 10510, 2007.

[18] Sudirjo F., Yani I., Hernawan M. A., Rukmana A. Y., and Nasution M. A., “Analysis of the Influence of Product Features, Price Perception, Brand and Customer Experience on Repurchase Intention of Smartphone Product,” *Innov*. *J*. *Soc*. *Sci*. *Res*., vol. 3, no. 3, pp. 3325–3333, 2023.

[19] Gupta S. and Lehmann D. R., “Customer lifetime value and firm valuation,” in *Customer Lifetime Value*, Routledge, 2013, pp. 87–110.

[20] Ateş M. A., Bloemhof J., Van Raaij E. M., and Wynstra F., “Proactive environmental strategy in a supply chain context: the mediating role of investments,” *Int*. *J*. *Prod*. *Res*., vol. 50, no. 4, pp. 1079–1095, 2012.

[21] Joung C. B., Carrell J., Sarkar P., and Feng S. C., “Categorization of indicators for sustainable manufacturing,” *Ecol*. *Indic*., vol. 24, pp. 148–157, 2013.

[22] Baah C. et al., “Examining the correlations between stakeholder pressures, green production practices, firm reputation, environmental and financial performance: Evidence from manufacturing SMEs,” *Sustain*. *Prod*. *Consum*., vol. 27, pp. 100–114, 2021.

[23] Kaiser F. G., Wölfing S., and Fuhrer U., “Environmental attitude and ecological behaviour,” *J*. *Environ*. *Psychol*., vol. 19, no. 1, pp. 1–19, 1999.

[24] Milfont T. L. and Duckitt J., “The structure of environmental attitudes: A first-and second-order confirmatory factor analysis,” *J*. *Environ*. *Psychol*., vol. 24, no. 3, pp. 289–303, 2004.

[25] Schultz P. W., Shriver C., Tabanico J. J., and Khazian A. M., “Implicit connections with nature,” *J*. *Environ*. *Psychol*., vol. 24, no. 1, pp. 31–42, 2004.

[26] Vining J. and Ebreo A., “An evaluation of the public response to a community recycling education program,” *Soc*. *Nat*. *Resour*., vol. 2, no. 1, pp. 23–36, 1989.

[27] Todaro N. M., Testa F., Daddi T., and Iraldo F., “The influence of managers’ awareness of climate change, perceived climate risk exposure and risk tolerance on the adoption of corporate responses to climate change,” *Bus*. *Strateg*. *Environ*., vol. 30, no. 2, pp. 1232–1248, 2021.

[28] Dunlap R. E., Van Liere K. D., Mertig A. G., and Jones R. E., “New trends in measuring environmental attitudes: measuring endorsement of the new ecological paradigm: a revised NEP scale,” *J*. *Soc*. *Issues*, vol. 56, no. 3, pp. 425–442, 2000.

[29] Fielding K. S., McDonald R., and Louis W. R., “Theory of planned behaviour, identity and intentions to engage in environmental activism,” *J*. *Environ*. *Psychol*., vol. 28, no. 4, pp. 318–326, 2008.

[30] da Silva P. C., de Oliveira Neto G. C., Correia J. M. F., and Tucci H. N. P., “Evaluation of economic, environmental and operational performance of the adoption of cleaner production: Survey in large textile industries,” *J*. *Clean*. *Prod*., vol. 278, p. 123855, 2021.

[31] Yang R., Tang W., and Zhang J., “Technology improvement strategy for green products under competition: The role of government subsidy,” *Eur*. *J*. *Oper*. *Res*., vol. 289, no. 2, pp. 553–568, 2021.

[32] Shrivastava P. and Hart S., “Creating sustainable corporations,” *Bus*. *Strateg*. *Environ*., vol. 4, no. 3, pp. 154–165, 1995.

[33] Chen Y.-S., Lai S.-B., and Wen C.-T., “The influence of green innovation performance on corporate advantage in Taiwan,” *J*. *Bus*. *ethics*, vol. 67, pp. 331–339, 2006.

[34] Peng H. and Liu Y., “A comprehensive analysis of cleaner production policies in China,” *J*. *Clean*. *Prod*., vol. 135, pp. 1138–1149, 2016.

[35] Sibarani L. and Genoveva G., “The Customer Pressure And Organizational Commitment On Environmental Performance Mediating Proactive Enviromental Strategies,” *Arch*. *Bus*. *Res*., vol. 7, no. 7, 2019.

[36] Bresciani S., Rehman S. U., Giovando G., and Alam G. M., “The role of environmental management accounting and environmental knowledge management practices influence on environmental performance: mediated-moderated model,” *J*. *Knowl*. *Manag*., vol. 27, no. 4, pp. 896–918, 2023.

[37] Daddi T., Testa F., Frey M., and Iraldo F., “Exploring the link between institutional pressures and environmental management systems effectiveness: An empirical study,” *J*. *Environ*. *Manage*., vol. 183, pp. 647–656, 2016. doi: 10.1016/j.jenvman.2016.09.025 27637805

[38] Amir M. and Chaudhry N. I., “Linking environmental strategy to firm performance: A sequential mediation model via environmental management accounting and top management commitment,” *Pakistan J*. *Commer*. *Soc*. *Sci*., vol. 13, no. 4, pp. 849–867, 2019.

[39] Burritt R. L., Hahn T., and Schaltegger S., “Towards a comprehensive framework for environmental management accounting—Links between business actors and environmental management accounting tools,” *Aust*. *Account*. *Rev*., vol. 12, no. 27, pp. 39–50, 2002.

[40] Schaltegger S. and Burritt R., *Contemporary environmental accounting*: *issues*, *concepts and practice*. Routledge, 2017.

[41] Scarpellini S., Valero‐Gil J., Moneva J. M., and Andreaus M., “Environmental management capabilities for a ‘circular eco‐innovation,’” *Bus*. *Strateg*. *Environ*., vol. 29, no. 5, pp. 1850–1864, 2020.

[42] Zhang X., Chu Z., Ren L., and Xing J., “Open innovation and sustainable competitive advantage: The role of organizational learning,” *Technol*. *Forecast*. *Soc*. *Change*, vol. 186, p. 122114, 2023.

[43] Barney J., “Firm resources and sustained competitive advantage,” *J*. *Manage*., vol. 17, no. 1, pp. 99–120, 1991.

[44] Baah C., Agyabeng-Mensah Y., Afum E., and Lascano Armas J. A., “Exploring corporate environmental ethics and green creativity as antecedents of green competitive advantage, sustainable production and financial performance: empirical evidence from manufacturing firms,” *Benchmarking An Int*. *J*., vol. 31, no. 3, pp. 990–1008, 2024.

[45] Porter M. E., “Porter, ME (1996). What Is Strategy? Harvard Business Review, 74 (6), 61–78,” *Harv*. *Bus*. *Rev*., 1996.10158474

[46] Huang J.-W., Li Y.-H., and Yen M.-T., “The relationship between green innovation and business performance-the mediating effect of Brand image,” *Mark*. *Rev*., vol. 13, no. 1, p. 89, 2016.

[47] Severo E. A., de Guimarães J. C. F., and Dorion E. C. H., “Cleaner production and environmental management as sustainable product innovation antecedents: A survey in Brazilian industries,” *J*. *Clean*. *Prod*., vol. 142, pp. 87–97, 2017.

[48] Oliveira T., Thomas M., Baptista G., and Campos F., “Mobile payment: Understanding the determinants of customer adoption and intention to recommend the technology,” *Comput*. *Human Behav*., vol. 61, pp. 404–414, 2016.

[49] Bai Y., Yin J., Yuan Y., Guo Y., and Song D., “An innovative system for promoting cleaner production: mandatory cleaner production audits in China,” *J*. *Clean*. *Prod*., vol. 108, pp. 883–890, 2015.

[50] Cudjoe D., Yuan Q., and Han M. S., “An assessment of the influence of awareness of benefits and perceived difficulties on waste sorting intention in Beijing,” *J*. *Clean*. *Prod*., vol. 272, p. 123084, 2020.

[51] Brammer S., Hoejmose S., and Marchant K., “Environmental management in SME s in the UK: Practices, pressures and perceived benefits,” *Bus*. *Strateg*. *Environ*., vol. 21, no. 7, pp. 423–434, 2012.

[52] Henri J.-F. and Journeault M., “Eco-control: The influence of management control systems on environmental and economic performance,” *Accounting*, *Organ*. *Soc*., vol. 35, no. 1, pp. 63–80, 2010.

[53] Hillary R., “Environmental management systems and the smaller enterprise,” *J*. *Clean*. *Prod*., vol. 12, no. 6, pp. 561–569, 2004.

[54] Simpson M., Taylor N., and Barker K., “Environmental responsibility in SMEs: does it deliver competitive advantage?,” *Bus*. *Strateg*. *Environ*., vol. 13, no. 3, pp. 156–171, 2004.

[55] Burritt R. L., Herzig C., and Tadeo B. D., “Environmental management accounting for cleaner production: The case of a Philippine rice mill,” *J*. *Clean*. *Prod*., vol. 17, no. 4, pp. 431–439, 2009.

[56] Saeidi S. P., Othman M. S. H., Saeidi P., and Saeidi S. P., “The moderating role of environmental management accounting between environmental innovation and firm financial performance,” *Int*. *J*. *Bus*. *Perform*. *Manag*., vol. 19, no. 3, pp. 326–348, 2018.

[57] Habib M. A., Bao Y., Nabi N., Dulal M., Asha A. A., and Islam M., “Impact of strategic orientations on the implementation of green supply chain management practices and sustainable firm performance,” *Sustainability*, vol. 13, no. 1, p. 340, 2021.

[58] Speer T. L., “Growing the green market,” *Am*. *Demogr*., vol. 19, pp. 45–50, 1997.

[59] Gualandris J. and Kalchschmidt M., “Customer pressure and innovativeness: Their role in sustainable supply chain management,” *J*. *Purch*. *Supply Manag*., vol. 20, no. 2, pp. 92–103, 2014.

[60] Hoffman A. J., *From heresy to dogma*: *An institutional history of corporate environmentalism*. Stanford University Press, 2001.

[61] Bohdanowicz P., “European hoteliers’ environmental attitudes: Greening the business,” *Cornell Hotel Restaur*. *Adm*. *Q*., vol. 46, no. 2, pp. 188–204, 2005.

[62] Chenhall R. H., “Management control systems design within its organizational context: findings from contingency-based research and directions for the future,” *Accounting*, *Organ*. *Soc*., vol. 28, no. 2–3, pp. 127–168, 2003.

[63] Chaudhry N. I., Asad H., and Hussain R. I., “Environmental innovation and financial performance: Mediating role of environmental management accounting and firm’s environmental strategy,” *Pakistan J*. *Commer*. *Soc*. *Sci*., vol. 14, no. 3, pp. 715–737, 2020.

[64] Lisi I. E., “Translating environmental motivations into performance: The role of environmental performance measurement systems,” *Manag*. *Account*. *Res*., vol. 29, pp. 27–44, 2015.

[65] Sharma S., “Managerial interpretations and organizational context as predictors of corporate choice of environmental strategy,” *Acad*. *Manag*. *J*., vol. 43, no. 4, pp. 681–697, 2000.

[66] Dibrell C., Craig J. B., and Hansen E. N., “How managerial attitudes toward the natural environment affect market orientation and innovation,” *J*. *Bus*. *Res*., vol. 64, no. 4, pp. 401–407, 2011.

[67] Appiah-Nimo C. and Chovancová M., “Improving firm sustainable performance: the role of market orientation,” in *Proceedings of the International Conference on Business Excellence*, 2020, vol. 14, no. 1, pp. 780–787.

[68] Agarwal R. and Helfat C. E., “Strategic renewal of organizations,” *Organ*. *Sci*., vol. 20, no. 2, pp. 281–293, 2009.

[69] Schaltegger S., Burritt R., Zvezdov D., Hörisch J., and Tingey‐Holyoak J., “Management roles and sustainability information. Exploring corporate practice,” *Aust*. *Account*. *Rev*., vol. 25, no. 4, pp. 328–345, 2015.

[70] Labodová A., “Implementing integrated management systems using a risk analysis based approach,” *J*. *Clean*. *Prod*., vol. 12, no. 6, pp. 571–580, 2004.

[71] Park E. and Kim K. J., “An integrated adoption model of mobile cloud services: exploration of key determinants and extension of technology acceptance model,” *Telemat*. *Informatics*, vol. 31, no. 3, pp. 376–385, 2014.

[72] Bennett M. D., Schaltegger S., and Zvezdov D., *Exploring corporate practices in management accounting for sustainability*. Icaew London, 2013.

[73] Armenti K. R., Moure-Eraso R., Slatin C., and Geiser K., “Primary prevention for worker health and safety: cleaner production and toxics use reduction in Massachusetts,” *J*. *Clean*. *Prod*., vol. 19, no. 5, pp. 488–497, 2011.

[74] Hicks C. and Dietmar R., “Improving cleaner production through the application of environmental management tools in China,” *J*. *Clean*. *Prod*., vol. 15, no. 5, pp. 395–408, 2007.

[75] Matos L. M. et al., “Implementation of cleaner production: A ten-year retrospective on benefits and difficulties found,” *J*. *Clean*. *Prod*., vol. 187, pp. 409–420, 2018.

[76] Eras J. J. C., Gutiérrez A. S., Lorenzo D. G., Martínez J. B. C., Hens L., and Vandecasteele C., “Bridging universities and industry through cleaner production activities. Experiences from the Cleaner Production Center at the University of Cienfuegos, Cuba,” *J*. *Clean*. *Prod*., vol. 108, pp. 873–882, 2015.

[77] Kjaerheim G., “Cleaner production and sustainability,” *J*. *Clean*. *Prod*., vol. 13, no. 4, pp. 329–339, 2005.

[78] Jansson Å., Nilsson F., and Rapp B., “Environmentally driven mode of business development: a management control perspective,” *Scand*. *J*. *Manag*., vol. 16, no. 3, pp. 305–333, 2000.

[79] Gerdin J. and Greve J., “Forms of contingency fit in management accounting research—a critical review,” *Accounting*, *Organ*. *Soc*., vol. 29, no. 3–4, pp. 303–326, 2004.

[80] Ambe C. M., “Environmental management accounting in South Africa: Status, challenges and implementation framework.” Tshwane University of Technology, 2007.

[81] Schaltegger S., Bennett M., Burritt R. L., and Jasch C., “Eco-efficiency in industry and science,” *Environ*. *Manag*. *Account*. *Clean*. *Prod*., vol. 5, 2010.

[82] Ferreira A., Moulang C., and Hendro B., “Environmental management accounting and innovation: an exploratory analysis,” *Accounting*, *Audit*. *Account*. *J*., 2010.

[83] Burritt R. L. and Schaltegger S., “Sustainability accounting and reporting: fad or trend?,” *Accounting*, *Audit*. *Account*. *J*., vol. 23, no. 7, pp. 829–846, 2010.

[84] Masanet-Llodra M. J., “Environmental management accounting: A case study research on innovative strategy,” *J*. *Bus*. *Ethics*, vol. 68, pp. 393–408, 2006.

[85] Cobra R. L. R. de B, Guardia M, Queiroz G. A, de Oliveira J. A, Ometto A R, and Esposto K. F, “‘Waste’ as the common ‘Gene’ connecting cleaner production and lean manufacturing: A proposition of a hybrid definition,” *Environ*. *Qual*. *Manag*., vol. 25, no. 1, pp. 25–40, 2015.

[86] Ozturk A. B., Nusair K., Okumus F., and Hua N., “The role of utilitarian and hedonic values on users’ continued usage intention in a mobile hotel booking environment,” *Int*. *J*. *Hosp*. *Manag*., vol. 57, pp. 106–115, 2016.

[87] Yüksel H., “An empirical evaluation of cleaner production practices in Turkey,” *J*. *Clean*. *Prod*., vol. 16, no. 1, pp. S50–S57, 2008.

[88] Alves S. M. and de Oliveira J. F. G., “Environmental adequacy of machining processes using Cleaner Production as strategy of environmental management,” *Production*, vol. 17, pp. 129–138, 2007.

[89] Saunders M., Lewis P., and Thornhill A., *Research methods for business students*. Pearson education, 2009.

[90] Creswell J. W. and Creswell J. D., *Research design*: *Qualitative*, *quantitative*, *and mixed methods approaches*. Sage publications, 2017.

[91] Chu S. H., Yang H., Lee M., and Park S., “The impact of institutional pressures on green supply chain management and firm performance: Top management roles and social capital,” *Sustainability*, vol. 9, no. 5, p. 764, 2017.

[92] Severo E. A., de Guimarães J. C. F., Dorion E. C. H., and Nodari C. H., “Cleaner production, environmental sustainability and organizational performance: an empirical study in the Brazilian Metal-Mechanic industry,” *J*. *Clean*. *Prod*., vol. 96, pp. 118–125, 2015.

[93] Lin Y.-H. and Chen Y.-S., “Determinants of green competitive advantage: the roles of green knowledge sharing, green dynamic capabilities, and green service innovation,” *Qual*. *Quant*., vol. 51, pp. 1663–1685, 2017.

[94] Wang S., Wang H., and Wang J., “Exploring the effects of institutional pressures on the implementation of environmental management accounting: Do top management support and perceived benefit work?,” *Bus*. *Strateg*. *Environ*., vol. 28, no. 1, pp. 233–243, 2019.

[95] Latan H., Jabbour C. J. C., de Sousa Jabbour A. B. L., Wamba S. F., and Shahbaz M., “Effects of environmental strategy, environmental uncertainty and top management’s commitment on corporate environmental performance: The role of environmental management accounting,” *J*. *Clean*. *Prod*., vol. 180, pp. 297–306, 2018.

[96] Book White, *White Book on Vietnam Enterprises*. Ha Noi: Statistics Publishing House, 2020.

[97] Wold H., “Model construction and evaluation when theoretical knowledge is scarce: Theory and application of partial least squares,” in *Evaluation of econometric models*, Elsevier, 1980, pp. 47–74.

[98] Podsakoff P. M., MacKenzie S. B., Lee J.-Y., and Podsakoff N. P., “Common method biases in behavioral research: a critical review of the literature and recommended remedies.,” *J*. *Appl*. *Psychol*., vol. 88, no. 5, p. 879, 2003. doi: 10.1037/0021-9010.88.5.879 14516251

[99] Bagozzi R. P., Yi Y., and Phillips L. W., “Assessing construct validity in organizational research,” *Adm*. *Sci*. *Q*., pp. 421–458, 1991.

[100] Hair J, Sarstedt M, Ringle C. M., and Gudergan S. P, *Advanced issues in partial least squares structural equation modeling*. Sage Publications, 2017.

[101] Hair J. F., Black W. C., Babin B. J., and Anderson R. E., “Multivariate data analysis: Pearson new international edition,” *Essex Pearson Educ*. *Ltd*., vol. 1, no. 2, 2014.

[102] Chin W. W., Marcolin B. L., and Newsted P. R., “A partial least squares latent variable modeling approach for measuring interaction effects: Results from a Monte Carlo simulation study and an electronic-mail emotion/adoption study,” *Inf*. *Syst*. *Res*., vol. 14, no. 2, pp. 189–217, 2003.

[103] Gosselin M., “The effect of strategy and organizational structure on the adoption and implementation of activity-based costing,” *Accounting*, *Organ*. *Soc*., vol. 22, no. 2, pp. 105–122, 1997.

[104] Bartolomeo M., Bennett M., Bouma J. J., Heydkamp P., James P., and Wolters T., “Environmental management accounting in Europe: current practice and future potential,” *Eur*. *Account*. *Rev*., vol. 9, no. 1, pp. 31–52, 2000.

[105] Hansen D. R. and Mowen M. M., “Management Accounting: Thomson South-Western,” 2005.

[106] Berrone P., Fosfuri A., Gelabert L., and Gomez‐Mejia L. R., “Necessity as the mother of ‘green’inventions: Institutional pressures and environmental innovations,” *Strateg*. *Manag*. *J*., vol. 34, no. 8, pp. 891–909, 2013.

[107] Christ K. L. and Burritt R. L., “Environmental management accounting: the significance of contingent variables for adoption,” *J*. *Clean*. *Prod*., vol. 41, pp. 163–173, 2013.

